# Effect of maternal oxytocin on umbilical venous and arterial blood flows during physiological-based cord clamping in preterm lambs

**DOI:** 10.1371/journal.pone.0253306

**Published:** 2021-06-17

**Authors:** Fiona J. Stenning, Graeme R. Polglase, Arjan B. te Pas, Kelly J. Crossley, Martin Kluckow, Andrew W. Gill, Euan M. Wallace, Erin V. McGillick, Corinna Binder, Douglas A. Blank, Calum Roberts, Stuart B. Hooper

**Affiliations:** 1 The Ritchie Centre, Hudson Institute of Medical Research, Melbourne, Australia; 2 Department of Obstetrics and Gynaecology, Monash University, Melbourne, Australia; 3 Division of Neonatology, Department of Paediatrics, Leiden University Medical Centre, Leiden, The Netherlands; 4 Department of Neonatalogy, Royal North Shore Hospital and University of Sydney, Sydney, New South Wales, Australia; 5 Centre for Neonatal Research and Education, The University of Western Australia, Perth, Western Australia, Australia; 6 Newborn Research, The Royal Women’s Hospital, Melbourne, Australia; 7 Department of Paediatrics, Monash University, Melbourne, Australia; State University of New York at Buffalo, UNITED STATES

## Abstract

**Background:**

Delayed umbilical cord clamping (UCC) after birth is thought to cause placental to infant blood transfusion, but the mechanisms are unknown. It has been suggested that uterine contractions force blood out of the placenta and into the infant during delayed cord clamping. We have investigated the effect of uterine contractions, induced by maternal oxytocin administration, on umbilical artery (UA) and venous (UV) blood flows before and after ventilation onset to determine whether uterine contractions cause placental transfusion in preterm lambs.

**Methods and findings:**

At ~128 days of gestation, UA and UV blood flows, pulmonary arterial blood flow (PBF) and carotid arterial (CA) pressures and blood flows were measured in three groups of fetal sheep during delayed UCC; maternal oxytocin following mifepristone, mifepristone alone, and saline controls. Each successive uterine contraction significantly (p<0.05) decreased UV (26.2±6.0 to 14.1±4.5 mL.min^-1^.kg^-1^) and UA (41.2±6.3 to 20.7 ± 4.0 mL.min^-1^.kg^-1^) flows and increased CA pressure and flow (47.1±3.4 to 52.8±3.5 mmHg and 29.4±2.6 to 37.3±3.4 mL.min^-1^.kg^-1^). These flows and pressures were partially restored between contractions, but did not return to pre-oxytocin administration levels. Ventilation onset during DCC increased the effects of uterine contractions on UA and UV flows, with retrograde UA flow (away from the placenta) commonly occurring during diastole.

**Conclusions:**

We found no evidence that amplification of uterine contractions with oxytocin increase placental transfusion during DCC. Instead they decreased both UA and UV flow and caused a net loss of blood from the lamb. Uterine contractions did, however, have significant cardiovascular effects and reduced systemic and cerebral oxygenation.

## Introduction

While a simple procedure, clamping the umbilical cord at birth is not necessarily an innocuous act. When umbilical cord clamping (UCC) occurs before the infant has commenced breathing, cardiac output decreases due to the loss of umbilical vein-dependent venous return to the heart [1]. Although an increase in pulmonary blood flow (PBF) takes over the role of providing venous return to the heart after birth [2], it can only do so after lung aeration [3]. Thus, following UCC, cardiac output remains reduced until after PBF has increased [1]. However, when UCC occurs after PBF has increased, pulmonary venous return immediately replaces umbilical venous return without any loss in cardiac output [1,4]. Encouraging the infant to commence breathing before UCC is called physiological based cord clamping (PBCC) [3].

In addition to maintaining cardiac output, delaying UCC for 60 seconds or more in preterm infants has been shown to reduce the risk of intraventricular haemorrhage, necrotising enterocolitis and late onset sepsis [5], although a recent meta-analysis has limited the benefit to reduced mortality [6]. Preterm infants receiving delayed UCC have increased haemoglobin and haematocrit values at 24 hours after birth and are less likely to require a blood transfusion [6]. In this context, delayed UCC is thought to provide a time-dependent placental to infant blood transfusion [7,8], but there is some question as to how this occurs [9]. It is also unclear what factors, particularly those associated with the routine management of the third stage of labour, could interfere with the physiological benefits of delayed UCC.

Active management of the third stage of labour reduces the risk of postpartum haemorrhage (PPH) and is comprised of three main components: maternal administration of a uterotonic (eg oxytocin) to contract the uterus, immediate UCC and controlled umbilical cord traction [10]. While delayed UCC of up to 60 seconds is now recommended for all infants not requiring resuscitation [11], it remains unclear when uterotonics should be administered during delayed UCC [12]. Current guidelines in many countries recommend that a uterotonic is administered at delivery of the infant’s anterior shoulder, which means that the uterotonic has the potential to alter placenta to infant transfusion if UCC is delayed. However, it is unknown whether uterotonics, and the associated uterine contractions, influence placental transfusion during delayed UCC.

Uterine contractions are commonly thought to increase the rate of placental transfusion by forcing blood out of the placenta into the infant [13]. However, this suggestion predates investigations showing that uterine contractions increase umbilical vascular resistance and reduce umbilical blood flows during labour in humans and cows [14,15] and following a positive oxytocin challenge test in humans [16]. Furthermore, the bidirectional umbilical artery flow observed during delayed UCC in infants, is thought to result from complete closure of umbilical vessels during a contraction after birth [17]. However, no studies have investigated the interaction between uterine contractions and fetal/neonatal cardiovascular function during PBCC and as such there is no clear guide for clinicians as to when a uterotonic should be administered. We hypothesised that maternal oxytocin administration would reduce UA and UV flows and adversely affect the carotid arterial pressure and blood flow changes during PBCC.

## Materials and methods

### Ethical approval

All experimental procedures were performed in accordance with the National Health and Medical Research Council (NHMRC) Code of Practice for the Care and Use of Animals for Scientific Purposes and were approved by the Monash Medical Centre ‘A’ Animal Ethics Committee, Monash University. Animals were sourced from the Monash University Animal Research Platform, Gippsland, Victoria, Australia and 16 pregnant Border-Leicester/Merino ewes were used in this experiment. Anaesthetic, euthanasia and surgical procedures are outlined below and are in accordance with the guidelines set out by Grundy, 2015. All ewes were fed *ad libitum* and had unrestricted access to water; animals were fasted for 12-24h before surgery.

#### Experimental protocol

To prematurely induce uterine contractions in response to oxytocin before term, ewes were given a 50mg intramuscular injection of the progesterone antagonist, mifepristone (RU486) 24-hours prior to delivery. Pregnant Border-Leicester/Merino ewes at 126 ± 1 days gestation (term ~ 147 days) were allocated to one of three experimental groups, 1) oxytocin plus mifepristone, 2) mifepristone alone and 3) saline control. Ewes in the oxytocin group had singleton pregnancies while ewes in the mifepristone and saline groups had either singleton or twin pregnancies. While mifepristone would be expected to eventually induce labour [18], no ewes were in labour at the time of delivery, as delivery occurred within 24 hrs after mifepristone injection.

Twenty-four hours after mifepristone administration, ewes were anesthetised initially with sodium thiopentone (Pentothal i.v.; 1g in 20 mL) and, following intubation, anaesthesia was maintained with inhaled isoflurane (1.5–3%) in air/oxygen. A hysterotomy was used to expose the head and chest of the lamb and polyvinyl catheters were inserted into the left carotid artery for pressure recording and blood sampling, and the left jugular vein for maintenance of anaesthesia. Ultrasonic flow probes were place on the lamb’s right carotid artery (CA) and the left main pulmonary artery. Ultrasonic flow probes were also placed around an UA and UV and a cuffed endotracheal tube was inserted into the trachea. The fetal forehead was shaved and a near infrared spectroscopy sensor (NIRS; Foresight CASMED, Branford CT) placed over the cortex to measure cerebral oxygenation and a pulse oximeter sensor (Masimo, Irvine CA) was placed on the right forelimb to measure arterial oxygen saturation. Baseline physiological parameters were recorded using LabChart (ADInstruments, Sydney, Australia) before starting any interventions. In mifepristone and oxytocin treated lambs, a known number of biotin-labelled red blood cells (RBCs) were injected into the lamb ~10 min before a venous blood sample was collected. This was used to measure the combined blood volume of the lamb and its placenta as previously described [19].

Ewes were placed into a lateral position and lambs were delivered onto a table next to the ewe with the umbilical cord intact, taking care to avoid any obstruction of umbilical blood flows; assessed from the UA and UV blood flow measurements. Following a stabilisation period, ewes in the oxytocin group were given an intravenous (i.v.) infusion of 10 IU oxytocin (Syntocinon), whereas ewes in the mifepristone alone and saline groups were given an equivalent volume of saline (i.v.). Approximately 5 minutes after giving the intravenous infusion, ventilation of the lamb commenced using an initial sustained inflation (35 cmH_2_O) for 30 seconds followed by volume-controlled positive pressure ventilation (Dräegar Babylog 8000+ ventilator, Dräegar, Lübeck, Germany) using a tidal volume (Vt) of 7.5 mL/kg and an end expiratory pressure (PEEP) of 5 cmH_2_O. The fraction of inspired oxygen (FiO_2_) and the respiratory rate were initially set at 21% and 60 breaths per minute, respectively, but were altered to maintain a target arterial oxygen saturation (SaO_2_) of 90–95% and an arterial carbon dioxide level (SaCO_2_) of 50–55 mmHg.

Lambs were ventilated for 30 minutes after ventilation onset and samples for blood gas analysis were collected at 5-minute intervals for the duration of the experiment. UCC occurred at >14 mins after ventilation onset to properly assess the ongoing effects of uterine contractions on umbilical blood flows after ventilation onset. Following UCC, the lamb received an intravenous infusion of alfaxane (5-10mg/kg/hr; Jurox, East Tamaki, Auckland, New Zealand) in 5% dextrose via the jugular vein to maintain anaesthesia for the remainder of the experiment. In addition, a venous blood sample was collected immediately before a known number of biotin-labelled red blood cells (RBCs) were injected into the lamb for a second time. A venous blood sample was again collected ~10 min later to measure the lamb’s blood volume, which was expressed as a percentage of the original lamb plus placental blood volume. Following the experiment, ewes and lambs were euthanized using an intravenous overdose injection of sodium pentobarbitone (100 mg/kg IV, Lethobarb, Virbac, Australia, Pty LTD).

### Statistical analyses

All physiological parameters were recorded in real time using LabChart (ADInstruments, NSW, Australia) and these data were analysed offline. A power analysis was based on the expected changes in UA and UV flow observed previously (Boere *et al*., 2014) and the variability in the reduction in UA and UV blood flow measured following ventilation onset (Blank *et al*., 2017). Values were averaged over ten heart beats every 30 seconds for 5 mins immediately after commencing the oxytocin/saline infusion and after ventilation onset and then at 5-minute intervals, ensuring that measurement points did not coincide with a contraction. In oxytocin administered lambs, values were averaged over five heartbeats immediately prior to and during each contraction, avoiding periods of recording disruption caused by blood sampling. Fetal baseline data and UV and UA flow reduction durations were analysed using a one-way ANOVA. Following treatment, the physiological data were analysed by time and group using a two-way repeated measures ANOVA with post hoc analysis (Holm-Sidak; Sigmastat v3.0, SPSS Inc.). Differences in UV, UA and CA flows and CA pressures measured immediately before and during each contraction in oxytocin administered animals were analysed by a paired t-test that included all contractions; no contractions were observed in mifepristone alone and saline groups and so only a time-based analysis was performed in these groups. Similarly, differences in percentage reduction in UV flow caused by contractions before and after ventilation onset were also compared using an unpaired t-test of all contractions combined. We accepted statistical significance at p < 0.05. Data is presented as mean ± standard error of the mean (SEM).

## Results

### Fetal & newborn characteristics

Fetal weights and blood gases measured before commencing the experiment were similar in all groups (Table 1). The time between ventilation onset and UCC in maternal oxytocin treated lambs was 16.4 ± 1.0 mins. During ventilation, the peak inflation pressure (PIP) required to achieve a set tidal volume, the respiration rate (Cont: 60.8 1.0: and mean airway pressure were similar between groups (Table 2), as were the arterial blood gas parameters (Table 2). The percentage of total blood volume (placenta plus lamb) remaining in the lamb following UCC tended to be less in oxytocin-treated compared to saline-treated (54.5 ± 0.2% vs 56.8 ± 0.2%) lambs but this was not significantly different from age-matched historical controls.

**Table 1:** Fetal characteristics.

	Oxytocin	RU486	Control
n (males)	5 (2)	5 (4)	5 (3)
Weight (kg)	3.17 ± 0.19	3.04 ± 0.14	3.07 ± 0.1
pH	7.21 ± 0.02	7.22 ± 0.03	7.26 ± 0.03
PaO_2_ (mmHg)	23.8 ± 1.94	23.86 ± 1.97	25.86 ± 2.43
PaCO_2_ (mmHg)	56.03 ± 2.4	56.18 ± 1.55	56.06 ± 6.12
SaO_2_ (%)	60.06 ± 6.88	60.16 ± 2.89	62.28 ± 6.59
Hb (g dl^-1^)	12.76 ± 0.51	12.48 ± 0.75	12.04 ± 0.83

**Table 2:** Arterial blood gas parameters.

*Time (mins)*	*Group*	*Mean Airway Pressure*	*Tidal Volume*	*Respiratory Rate*	*PaO*_*2*_	*PaCO*_*2*_	*SaO*_*2*_	*AaO*_*2*_
***5***	***Oxytocin***	19.8 ± 1.3	5.6 ± 0.8	58.3 ± 0.3	19.5 ± 3.4	62.4 ± 5.9	56.9 ± 6.4	258 ± 57
	***RU486***	16.4 ± 0.9	6.9 ± 0.3	61.4 ± 2.9	32.4 ± 9.2	63.1 ± 3.8	63.8 ± 10.7	258 ± 41
	***Control***	17.3 ± 2.4	5.2 ± 0.3	59.4 ± 1.2	20.7 ± 3.4	64.2 ± 6.1	51.3 ± 9.7	463 ± 44
***10***	***Oxytocin***	19.3 ± 1.3	6.4 ± 0.6	58.3 ± 0.3	32.8 ± 4.1	53.5 ± 6.1	79.6 ± 5.7	237 ± 53
	***RU486***	15 ± 0.7	6.8 ± 0.3	57.0 ± 1.1	39.7 ± 6.8	52 ± 5.2	88.8 ± 3.6	308 ± 49
	***Control***	14.9 ± 0.7	5.6 ± 0.4	59.4 ± 1.3	32.3 ± 5.3	68.5 ± 9.9	82.5 ± 5.2	356 ± 56
***20***	***Oxytocin***	19.5 ± 1.0	6.6 ± 0.7	58.3 ± 0.3	34.1 ± 5.5	52.31 ± 5.7	78.7 ± 6.7	272 ± 51
	***RU486***	15.4 ± 1.3	6.7 ± 0.4	59.8 ± 3.9	40.4 ± 4.4	51.4 ± 5.7	91.7 ± 2.6	325 ± 62
	***Control***	16.6 ± 2.3	5.6 ± 0.3	61.7 ± 0.8	40.6 ± 3.4	65.5 ± 6.0	86.9 ± 1.3	489 ± 46
***30***	***Oxytocin***	18.5 ± 1.3	6.8 ± 0.8	58.3 ± 0.3	30.2 ± 2.2	48.0 ± 8.9	80.5 ± 3.8	313 ± 41
	***RU486***	14.8 ± 1.2	7.1 ± 0.3	64.6 ± 3.9	34.7 ± 3.3	52.3 ± 4.9	84.2 ± 2.6	258 ± 37
	***Control***	17.0 ± 2.2	5.7 ± 0.3	61.0 ± 1.1	44.6 ± 5.4	67.2 ± 7.0	87.5 ± 3.0	494 ± 56

### Cardiovascular changes prior to ventilation onset

**Fig 1:**
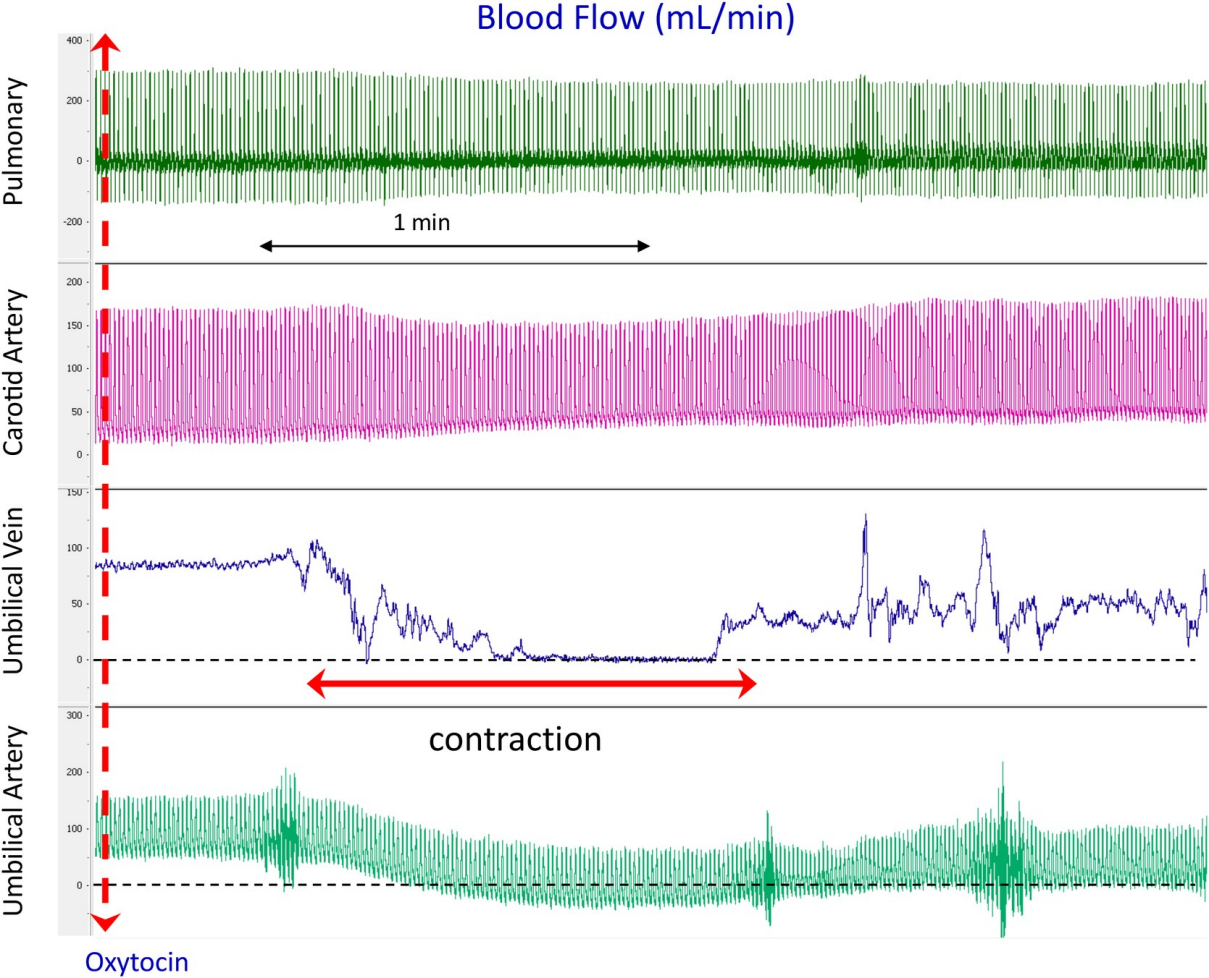
Physiological recordings of blood flows in the left pulmonary artery (PA), carotid artery, umbilical vein (UV) and umbilical artery (UA) before and immediately after oxytocin administration (iv) to the ewe in a newborn lamb prior to UCC and before ventilation onset. Note that during the contraction, UV flow decreases to 0, UA flow decreases and reverses during diastole (indicated by diastolic flows below zero) and CA flow increases.

**Fig 2:**
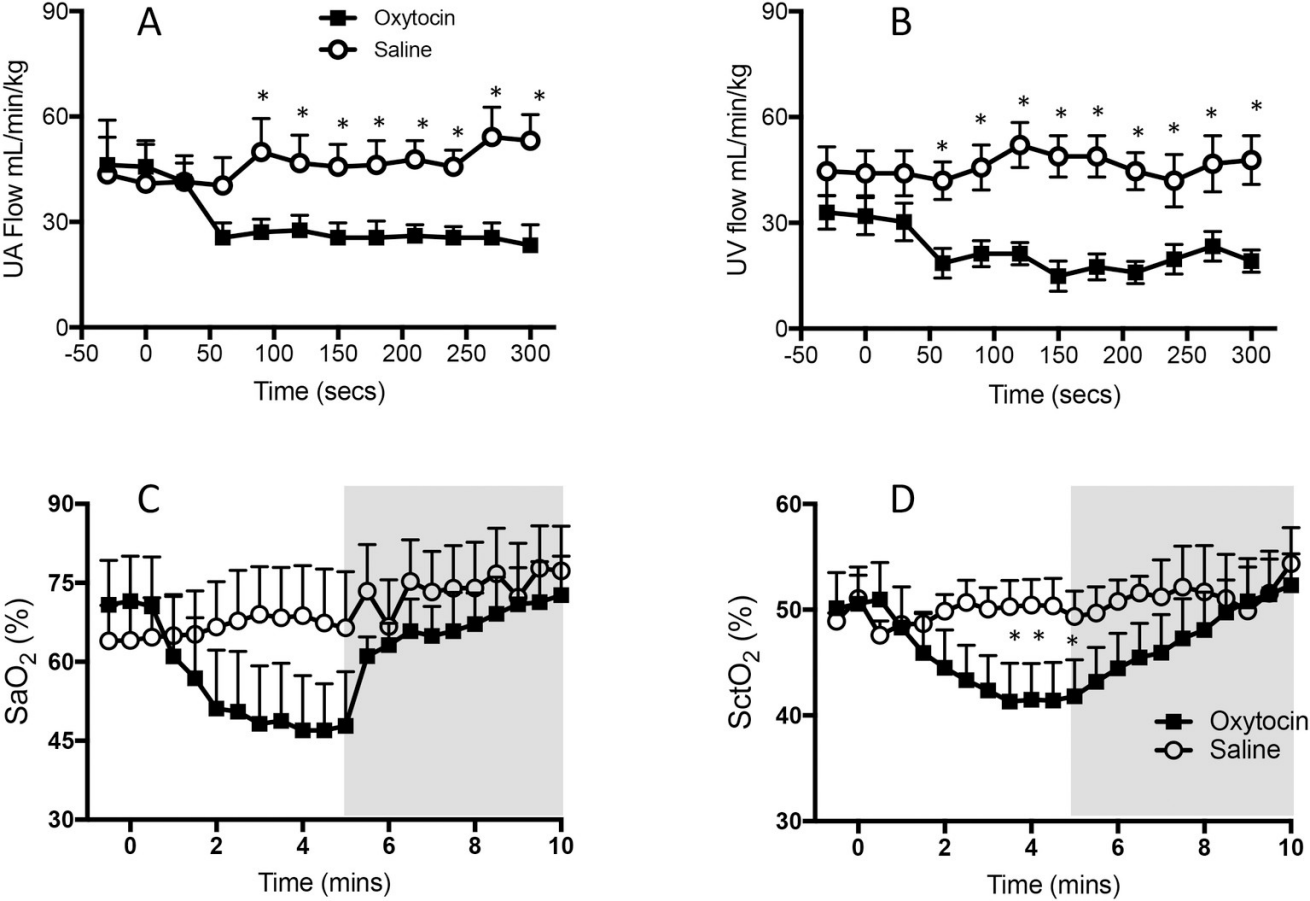
Changes in (A) mean (± SEM) umbilical arterial (UA) blood flow, (B) venous (UV) blood flow (C) arterial oxygen saturation (SaO_2_) and (D) cerebral tissue oxygenation (SctO_2_) levels in RU486 plus saline treated lambs (open cycles) and RU486 plus oxytocin treated lambs (closed squares). Samples were collected during the first 5 minutes after oxytocin/saline administration and before ventilation onset (A & B) and for 5 minutes after ventilation onset (indicated by shaded area; C & D). Data from saline-infused control animals (no RU486) have been omitted for clarity.

Maternal oxytocin resulted in uterine contractions within a median of 70 seconds (range 57–98 sec). Each contraction resulted in significant reductions in UA and UV blood flows as well as increases in CA pressure and blood flows (Figs 1 & 2). As saline infusions into control and mifepristone treated ewes resulted in no contractions and cardiovascular changes (Fig 2), the presented results mainly report cardiovascular parameters before and after oxytocin-induced uterine contractions.

#### Umbilical venous flow

**Fig 3:**
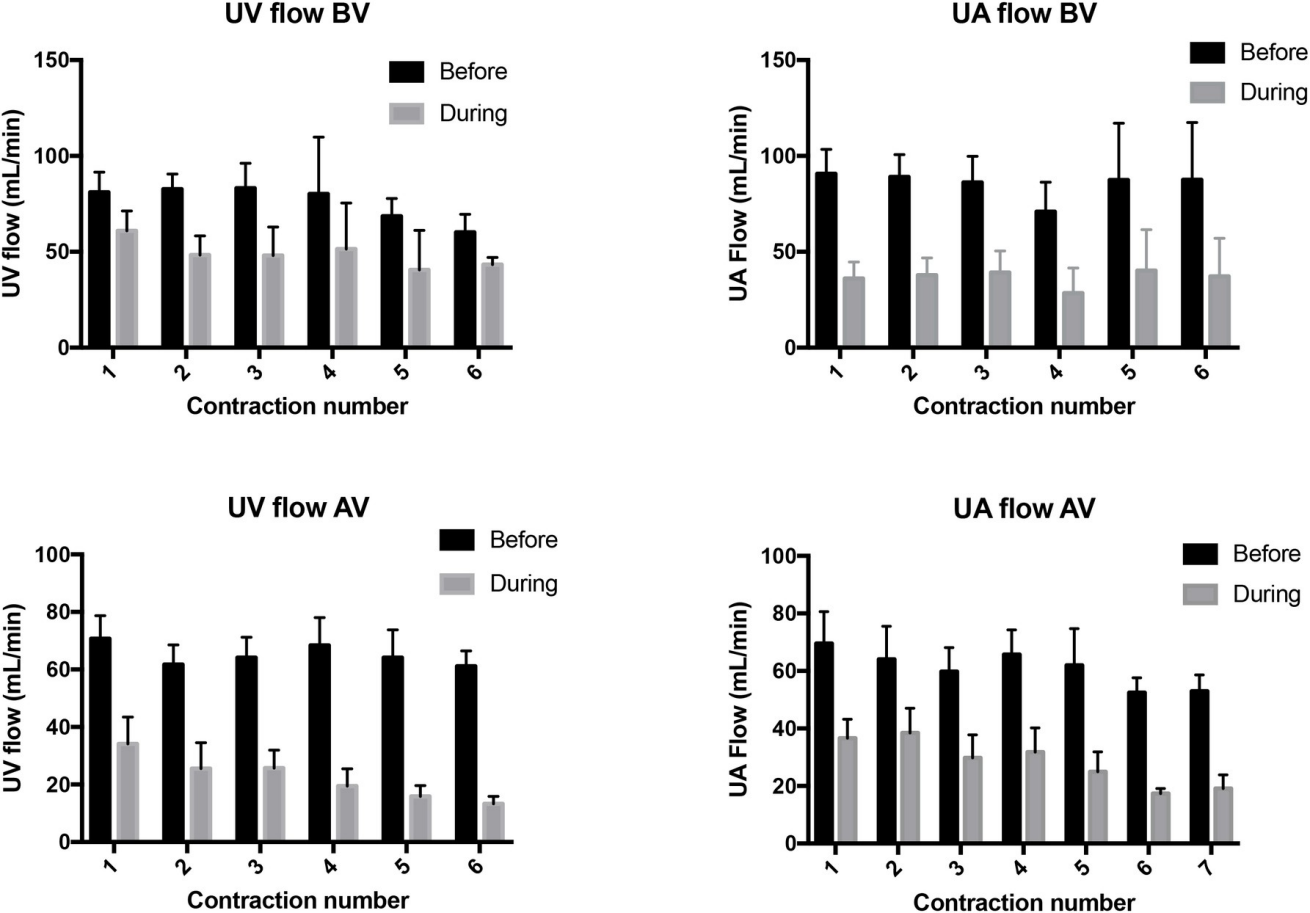
Blood flow in the umbilical vein (UV) and umbilical artery (UA) measured immediately before (black bar) and during (grey bar) consecutive uterine contractions and before (BV) and after (AV) ventilation onset. UV and UA blood flows measured during each contraction were significantly lower than flows measured immediately prior to the contraction (p<0.0001).

Maternal oxytocin administration reduced (p<0.005) UV flow from 26.2 ± 6.0 mL min^-1^ kg^-1^ at the start of the first contraction to 14.1 ± 4.5 mL min^-1^ kg^-1^ at the end of the first contraction (Figs 1 & 3). While UV flow increased between each contraction, the flow measured between contractions remained below the pre-oxytocin administration level at all times during the remainder of the experiment (Fig 2). With each consecutive contraction, UV flow was significantly reduced before increasing again following the contraction (p<0.0001). Relative to UV flow before each contraction, UV flow was reduced by 39.0 ± 6.5%, 24.5 ± 9.4%, 43.7 ± 7.4%, 45.5 ± 10.4%, 40.3 ± 3.5% and 45.6 ± 12.1% during the first 6 contractions, respectively (Fig 3).

#### Umbilical arterial flow

Maternal oxytocin administration reduced mean UA flow from 41.2 ± 6.3 mL min^-1^ kg^-1^ to 20.7 ± 4.0 mL min^-1^ kg^-1^ following the first contraction (Figs 1 & 3), which remained reduced for the remainder of the experiment (p<0.05). While mean UA flow increased between each contraction, each successive contraction caused a reduction in UA flow (p<0.0001); UA flow was reduced (p<0.05) by 45.5 ± 4.3%, 61.9 ± 7.1%, 59.2 ± 7.5%, 57.0 ± 7.8%, 63.2 ± 8.6% and 57.0 ± 8.2% by the 1^st^, 2^nd^, 3^rd^, 4^th^, 5^th^ and 6^th^ contraction, respectively (Fig 3).

**Fig 4:**
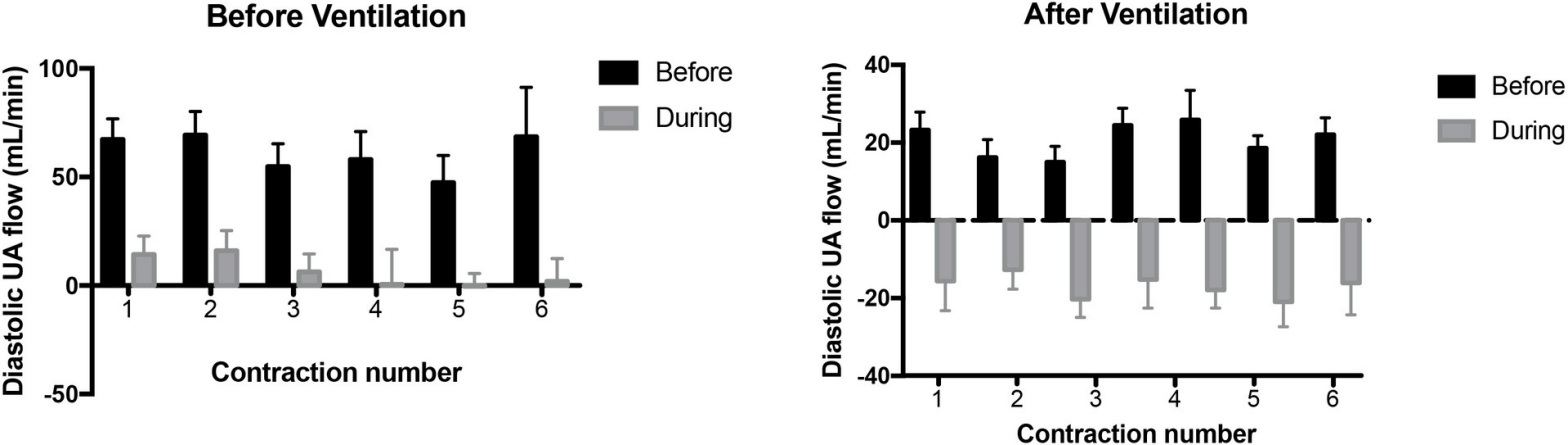
Diastolic blood flow in the umbilical artery (UA) measured immediately before (black bar) and during (grey bar) consecutive uterine contractions and before (BV) and after (AV) ventilation onset. Flows that have a value below zero indicate retrograde flow in the UA. Blood flows measured during each contraction were significantly lower than flows measured immediately prior to the contraction (p<0.0001).

Diastolic UA flow was reduced (p<0.0001) with each contraction, decreasing by 79.3 ± 12.2% with the first contraction and then by 82.1 ± 11.4%, 82.3 ± 12.6%, 101.4 ± 16.1%, 108.3 ± 19.9% and 107.1 ± 9.5% by the 2^nd^, 3^rd^, 4^th^, 5^th^ and 6^th^ contraction, respectively (Figs 1 & 4). A value greater than 100% indicates that blood flow in the UA was retrograde.

#### Umbilical venous-arterial flow

**Fig 5:**
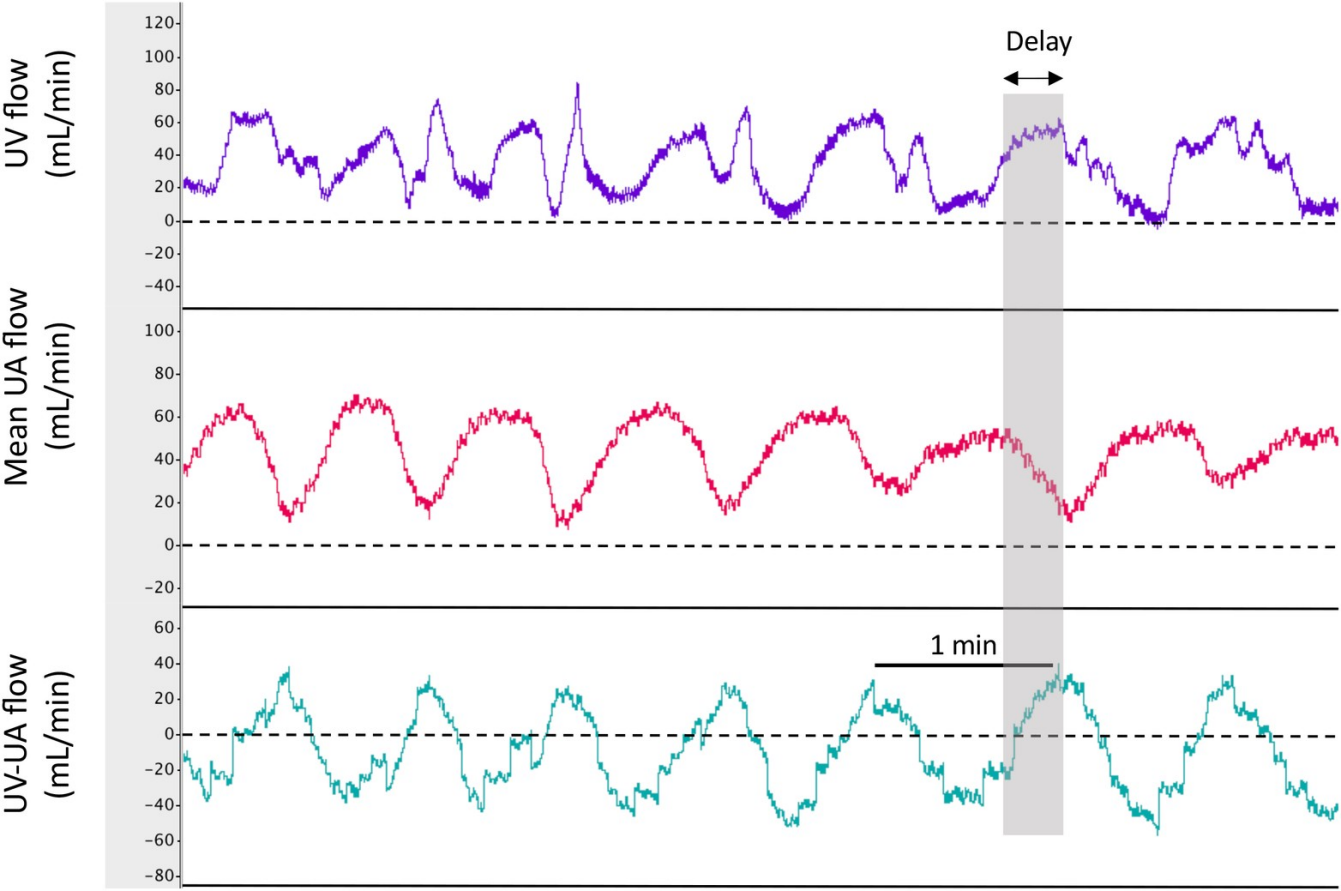
Physiological recordings of mean blood flows in the umbilical vein (UV), umbilical artery (UA) and the arithmetic derivation of UV-UA flow in a newborn lamb during consecutive uterine contractions. A value for UV-UA flow that is below zero indicates a net loss of blood flow from the lamb into the placenta. Note that during the contraction, both UV and UA flow decrease to near zero, but that the reduction in UV flow is significantly delayed compared to the UA flow. As a result, much of the reduction in UV flow occurs while the UA flow is recovering, resulting in a net loss of blood from the lamb. However, with the onset of the next contraction, because the reduction in UV flow is delayed, there is a net flow of blood from the placenta to the lamb.

Instantaneous UV-UA flow varied markedly between lambs depending upon how synchronous the contraction-induced reductions in UA and UV flow were. Fig 5 illustrates an example of flow reductions that were markedly asynchronous, resulting in an oscillating pattern of net blood accumulation (positive UV-UA value) and blood loss from the lamb (negative UV-UA value). The integral of the UV-UA flow over the period from oxytocin administration to ventilation onset, showed a net loss (-9.2 ± 3.5 mL/kg) of blood from the lamb over this period. This net loss was largely because the contraction-induced reduction in flow was significantly shorter in the UA than UV (24.5 ± 0.7 vs 32.3 ± 1.2 secs/contraction; p<0.05).

#### Heart rate, carotid arterial (CA) pressure and blood flow

**Fig 6:**
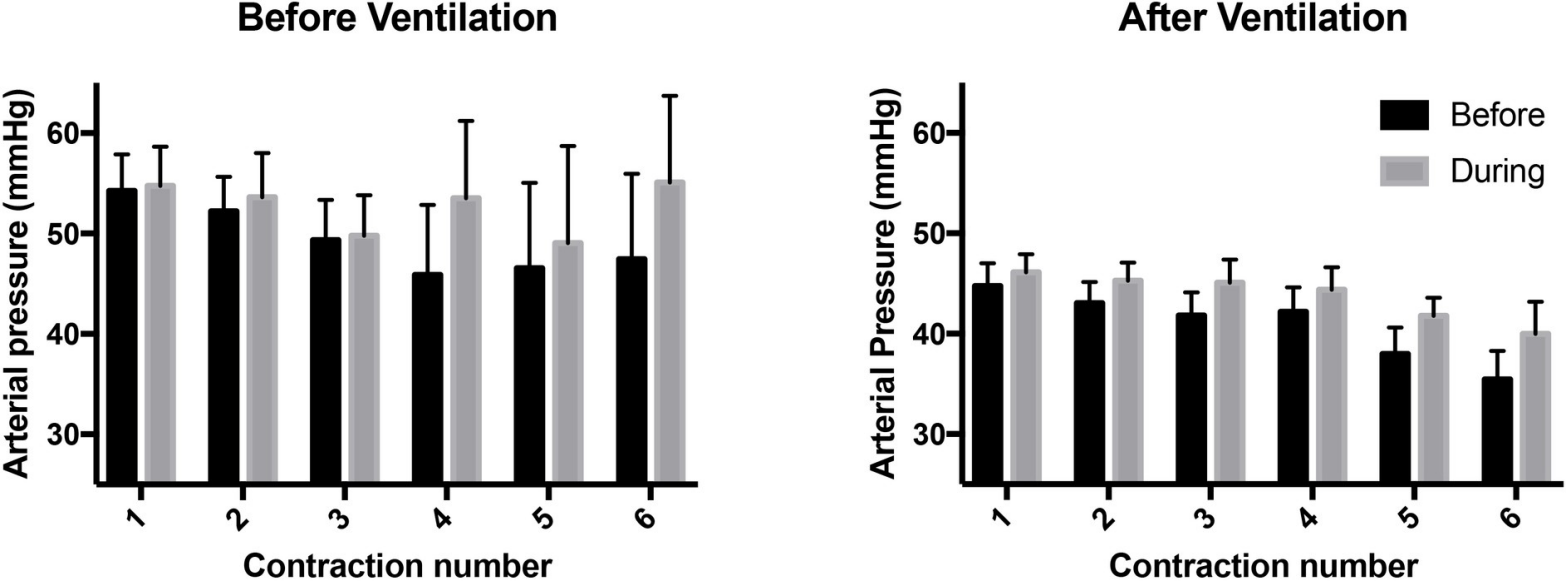
Mean carotid arterial (CA) pressures measured immediately before (black bar) and during (grey bar) consecutive uterine contractions and before (BV) and after ventilation (AV) onset. Arterial pressures measured during each contraction were significantly higher than pressures measured immediately prior to the contraction (p<0.0002).

Maternal oxytocin administration significantly (p<0.05) increased CA pressure and flow in lambs from 47.1 ± 3.4 mmHg and 29.4 ± 2.6 mL min^-1^ kg^-1^ at 30 seconds prior to administration, to 52.8 ± 3.5 mmHg at 120 seconds and 37.3 ± 3.4 mL min^-1^ kg^-1^ at 240 seconds after oxytocin administration, respectively. Furthermore, heart rate (HR) increased from 154 ± 12 BPM prior to oxytocin administration, to 176 ± 15 at 150 seconds and 206 ± 12 BPM at 300 seconds after oxytocin administration (p<0.05). Before ventilation onset, CA pressure and flow increased (p<0.0002) during contractions (Fig 6).

#### Arterial (SaO_2_) and Cerebral oxygenation (SctO_2_)

Maternal oxytocin administration significantly reduced (p<0.05) arterial oxygen saturation (SaO_2_) in lambs from 67.7 ± 8.1% before the start of the first contraction to 49.1 ± 6.5% following the fourth contraction (Fig 2). SaO_2_ did not return to pre-oxytocin levels until after ventilation onset. SaO_2_ was not changed in the RU486 and control groups following saline administration and prior to ventilation onset. Maternal oxytocin administration also significantly reduced (p<0.05) cerebral oxygenation (SctO_2_) from 48.2 ± 4.1% before the first contraction to 40.0 ± 4.2% at the end of the third contraction. SctO_2_ did not return to pre-oxytocin administration levels until after ventilation onset (Fig 2).

### Cardiovascular changes following ventilation onset

**Fig 7:**
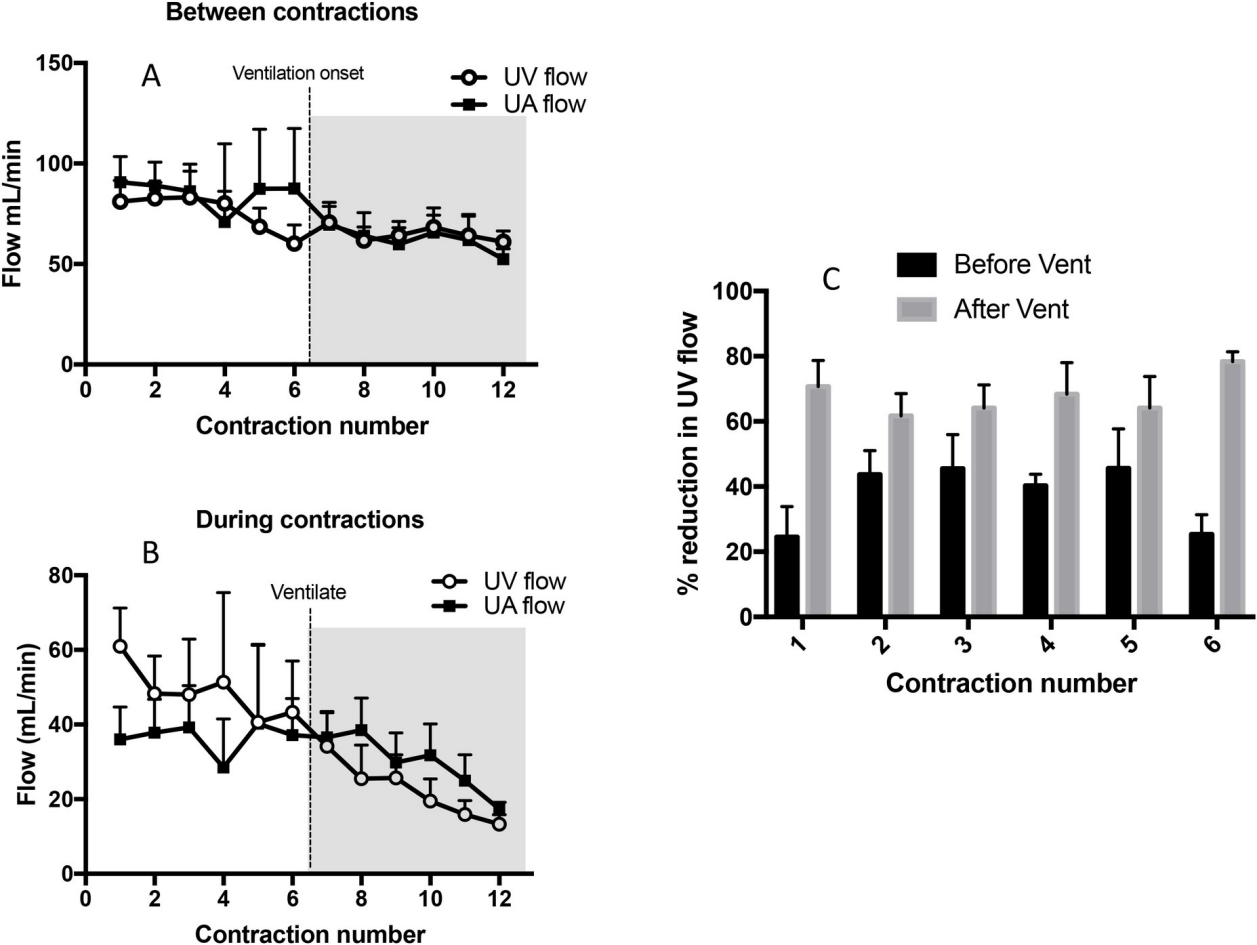
Mean blood flows in the umbilical vein (UV; open circles) and umbilical artery (UA; closed squares) measured immediately before (A) and during (B) consecutive uterine contractions before and after ventilation onset. Both UV and UA flow significantly decreased with increasing contraction number during (p<0.0001 and p = 0.015, respectively) between (p = 0.0001 and p = 0.001, respectively) contractions (C) The percentage reduction in UV flow during a uterine contraction was significantly greater (p<0.0001) after ventilation onset (grey bar) compared to before ventilation onset (black bar).

At the onset of ventilation, both UV and UA flow were significantly lower in lambs whose mothers were given oxytocin (measured between contractions) compared with those whose mothers were given saline and saline plus mifepristone (p<0.05). At 180 secs after ventilation onset, UA and UV flow were significantly reduced (p<0.05) in all groups, although the reduction was particularly marked during a contraction (Figs 1, 3, 4 & 7). Ventilation onset significantly increased SaO_2_ and SctO_2_ from 49.1 ± 6.5% and 40.0 ± 4.2% to 64.1 ± 4.4 and 44.0 ± 1.5, respectively in mifepristone and oxytocin administered lambs (Fig 2).

#### Umbilical venous flow

In maternal oxytocin administered animals, following ventilation onset, uterine contractions significantly reduced UV blood flow with each contraction (p<0.0001), which increased again between contractions (Fig 3). UV blood flow was reduced by 70.7 ± 8.0%, 61.7 ± 6.8%, 64.2 ± 7.0%, 68.4 ± 9.7%, 64.1 ± 9.6%, 78.3 ± 3.0% by the 1^st^, 2^nd^, 3^rd^, 4^th^, 5^th^ and 6^th^ contraction after ventilation onset, respectively. The percentage reductions in UV flow after ventilation onset were greater than the contraction-induced reductions in UV flow before ventilation onset (Fig 7).

#### Umbilical artery flow

In maternal oxytocin administered animals, following ventilation onset, uterine contractions reduced UA blood flow with each contraction (p<0.0001), which increased again between contractions (Fig 3). UA blood flow was reduced by 44.2 ± 9.7%, 43.0 ± 6.5%, 54.5 ± 6.3%, 54.3 ± 8.2%, 61.2 ± 3.9% and 65.4 ± 5.2% by the 1^st^, 2^nd^, 3^rd^, 4^th^, 5^th^ and 6^th^ contraction after ventilation onset, respectively.

Following ventilation onset, uterine contractions resulted in reversed or retrograde UA flow during diastole (Fig 4). Indeed, uterine contractions reduced UA flow during diastole by 232 ± 76%, 296 ± 121%, 349 ± 124%, 216 ± 64%, 297 ± 99%, 279 ± 83% by the 1^st^, 2^nd^, 3^rd^, 4^th^, 5^th^ and 6^th^ contraction, respectively; a percentage decrease >100% reflects retrograde flow away from the placenta.

#### Umbilical venous–umbilical artery flow

After ventilation onset, the pattern in instantaneous UV-UA flow (Fig 5) continued to vary markedly between lambs. The integral of the UV-UA flow over the period from ventilation onset until UCC, again demonstrated a net loss (-9.0 ± 3.8 mL/kg) of blood from the lamb over this period. However, as this period was significantly longer than the pre-ventilation period (16.4 ± 1.0 vs 5.1 ± 0.8 mins; p<0.05) this reflects a much lower blood loss rate. As observed prior to ventilation onset, the contraction-induced reduction in flow during individual contractions was significantly shorter in the UA than in the UV (30.4 ± 0.8 vs 39.8 ± 1.3 secs, respectively; p<0.05). These reductions in flow were longer following ventilation onset than before ventilation onset; 24.5 ± 0.7 vs 30.4 ± 0.8 secs in the UA (p<0.05) and 32.3 ± 1.2 vs 39.8 ± 1.3 secs in the UV (p<0.05).

#### Carotid artery pressure (P_CA_) and carotid artery blood flow (CaBF)

CA pressure decreased significantly in all three groups by 30 seconds after ventilation onset and was significantly higher in the oxytocin group compared to the control group at 30, 60, 120 and 150 seconds after ventilation onset. Following ventilation onset, each contraction significantly increased the mean CA pressure (p<0.0001) by 3.7 ± 3.6%, 5.7 ± 3.4%, 8.3 ± 3.9%, 5.4 ± 1.5%, 11.6 ± 6.2% and 12.7 ± 2.2% during the 1^st^, 2^nd^, 3^rd^, 4^th^, 5^th^ and 6^th^ contraction, respectively (Fig 6). Similarly, following ventilation onset, each contraction significantly increased CA blood flow (p<0.0001) by 22.7 ± 19%, 17.7 ± 12.7%, 21.2 ± 11.2%, 17.1 ± 10.1%, 31.6 ± 17.4% and 16.3 ± 5.3% during the 1^st^, 2^nd^, 3^rd^, 4^th^, 5^th^ and 6^th^ contraction, respectively.

## Discussion

While placental transfusion is thought to occur during delayed UCC, the underlying physiological mechanisms that cause a net shift of blood from the placenta into the infant are unknown [9,20]. However, it is important to understand the mechanisms involved, mainly to avoid factors that may retard or reverse placental transfusion. It has been proposed that uterine contractions “squeeze” blood out of the placenta into the infant [13], but this suggestion is contrary to the known effects of uterine contractions on umbilical blood flow [14–16]. We hypothesised that maternal oxytocin administration would reduce both umbilical artery and venous blood flows and compromise the cardiovascular transition during PBCC. We found that uterine contractions, induced by exogenous maternal oxytocin administration, rapidly and significantly reduced oxygen saturations and both UA and UV flows. While flow was partially restored between contractions (albeit below pre-oxytocin levels), each subsequent contraction markedly reduced UA and UV flows, particularly after ventilation onset when the duration of the flow reduction increased. Each contraction was also associated with increases in CA pressure and blood flow and reduced SaO_2_ and SctO_2_ levels (Figs 1 & 6), as has been shown to occur during cord “milking” or in response to UCC [1,21]. No contractions or changes in UA and UV flows were observed in saline-infused control and mifepristone treated animals before ventilation onset.

Our results clearly show that uterine contractions do not increase blood flow out of the placenta into newborn lambs during delayed UCC. Instead, uterine contractions markedly reduce both UA and UV blood flow, with the reductions progressively increasing with each successive uterine contraction, particularly after ventilation onset. This is consistent with the known effects of uterine contractions on UA and UV flows before birth in humans [14,16] and cows [15]. It is unknown why UA and UV flows did not return to pre-oxytocin levels between contractions, although an increase in basal uterine smooth muscle tone is a possible explanation, particularly as RU486 was used to block the inhibitory effect of progesterone on uterine activity. Nevertheless, the instantaneous reduction in UA and UV flow is likely due to mechanical compression of the intra-placental umbilical vessels as previously suggested [22].

An analysis of the instantaneous difference in UV and UA flows (UV-UA) (Fig 5) showed that during contractions there was a net loss of blood volume from the lamb, but during the relaxation phase, blood tended to shift back into the lamb (Fig 5). However, as the reduction in flow was significantly longer (by 5–6 secs) in the UV than in the UA, lambs tended to lose blood into the placenta in response to contractions. At the very least, this confirms that during a contraction, blood is not “squeezed” out of the placenta. By integrating the UV-UA difference over time, we calculated that lambs had lost 18.2 ± 6.3 mL/kg of blood into the placenta over the 16.4 ± 1.0 mins of DCC (i.e. both before and after ventilation onset). While this absolute volume change may not be accurate, because we only measured flow in one of two UAs and UVs in each lamb, we consider it likely that the flow pattern was similar in both sets of vessels. In addition, this calculation is consistent with our finding of a tendency for reduced blood volumes (% of total) in mifepristone plus oxytocin treated lambs compared to historical controls [23]. It is also consistent with the Cochrane Review showing that active (vs expectant) management of the 3^rd^ stage of labour significantly reduces birth weight in infants [10].

While the reduction in UV flow was longer than the reduction in UA flow, the instantaneous differential in UV and UA flow was complex. In some lambs, the reduction in UV flow was delayed, compared to the reduction in UA flow, by up to 20 seconds (Fig 5). This delay was likely due to the relatively high compliance and low pressures downstream within the fetal venous circulation, which delayed the measurement of the contraction-induced reduction in UV flow upstream in the placenta. In contrast, in the UA, a contraction-induced increase in resistance in the placenta downstream of the point of blood flow measurement causes an almost instantaneous decrease in UA flow. While the delay between the UA and UV flow decrease varied considerably between lambs, when it was highly asynchronous, it produced a contraction-induced cyclic pattern in the instantaneous UV-UA flow differential (Fig 5). At the very least, strong uterine contractions complicate the net movement of blood into and out of the newborn lamb and may influence the net volume of placental transfusion in infants. For instance, clamping the cord during or towards the end of a contraction may reduce the volume of blood entering the newborn, whereas clamping towards the end of the relaxation phase or at the beginning of the contraction may enhance blood transfer into the newborn. Perhaps this should be considered in future delayed UCC studies to achieve a more consistent degree of placental transfusion between infants. Nevertheless, considering that the influence of uterine contractions on umbilical blood flows has been known for decades, future RCTs should consider the potential effect of uterotonics on placental transfusion during delayed UCC [24].

Immediate UCC before ventilation onset results in a rapid (within 4 heart beats) increase (by 30%) in arterial pressure. This is due to an increase in systemic arterial resistance (afterload) caused by removal of the low resistance placental vascular bed from the systemic circulation and results in a pressure related increase in cerebral blood flow [1]. However, when ventilation precedes UCC (PBCC), this increase in arterial pressure and cerebral blood flow is reduced, as is the reduction in SctO_2_ and SaO_2_ associated with UCC [1,25]. The finding that each contraction resulted in an increase in CA pressure and flow is consistent with a contraction-induced increase in downstream resistance in umbilical arteries, which is similar to immediate UCC and cord milking [1,21].

A recent study demonstrated no difference in the net placental transfusion at vaginal birth in term infants when 10IU of exogenous oxytocin was administered intravenously immediately following delivery when compared with administration following cord clamping at 3 minutes [26]. Vain et al looked specifically at the role of oxytocin in placental transfusion and their findings are consistent with the findings of this study. While we could find no evidence for uterotonics increasing placental transfusion during delayed UCC, a previous study has suggested that maternal methylergometrine increases blood transfer rates to the infant during delayed UCC, without affecting the total amount transferred [13]. However, their conclusion that blood volume accumulated faster following methylergometrine was based on one time point being “more significant” in the methylergometrine group without any statistical comparison between the groups[13]. Furthermore, they [13] argued that previously measured high pressures (15–50 mmHg) within the umbilical vein during delayed UCC [27] would provide a pressure gradient for blood to move from the placenta into the infant during a contraction. However, they assumed that the pressure increase was caused by uterine contractions, despite the fact they were not measured or even commented on in the cited study [27]. Instead, that study showed the strong influence of breathing, crying and forced expirations on pressure changes in the umbilical vessels and that methylergometrine caused umbilical arteries to constrict, which is consistent with the findings of this study [27].

In view of the reductions in umbilical artery and venous blood flows, it is not surprising that oxytocin-induced uterine contractions caused a significant reduction in SaO_2_ and SctO_2_ during delayed UCC. Previous research has shown that non-labour uterine contractions, or Braxton Hicks contractions, cause a reduction in uterine artery blood flow and a transient reduction in placental oxygenation, which results in a transient fall in cerebral oxygenation (Oosterhof 1992, Sinding, 2016). Thus, strong uterine contractions in response to early oxytocin administration may explain why reductions in SaO_2_ can be observed during delayed UCC, particularly if the infant hasn’t established effective pulmonary ventilation. As such, the ability of delayed UCC to maintain SaO_2_ levels in infants prior to lung aeration will likely depend upon the presence/absence of uterine contractions. Nevertheless, as the initiation of respiratory support improved oxygenation in lambs, this emphasizes the importance of establishing pulmonary ventilation during delayed UCC (also known as PBCC) to stabilise oxygenation of the fetus during transition.

We administered mifepristone, a progesterone receptor antagonist, to prematurely reduce progesterone dominance of the sheep uterus and make it more sensitise to oxytocin. As this progesterone dominance renders the sheep uterus relatively quiescent until late in gestation, even with mifepristone, we required a high dose of oxytocin to simulate labour-like episodic uterine contractions. Typically, at caesarean section 5IU of exogenous oxytocin is given intravenously or at vaginal birth 10IU of exogenous oxytocin is administered intramuscularly. While mifepristone can induce labour in sheep, which takes more than 30hs [18], no ewes were in labour before the experiment and no coordinated uterine contractions occurred prior to oxytocin administration. As such we consider that the contractile response we elicited in this study was labour-like, but likely to be less than the response that would occur following a similar dose given during the 3^rd^ stage of labour. Furthermore, we would expect that the biological effects of our single dose of oxytocin would gradually diminish with time. We included a mifepristone control group (no oxytocin) because, in our preliminary studies, maternal mifepristone administration appeared to elevate fetal CA pressure and flow. As mifepristone crosses the placenta and increases fetal aldosterone levels, it is possible that the initial higher blood pressure resulted from increased aldosterone levels. Nevertheless, mifepristone in the absence of oxytocin had no effect on UA and UV flows and did not induce uterine contractions that were associated with reductions in UA and UV flows.

In order to observe the physiological changes following exogenous oxytocin administration and ventilation onset, the period of delayed UCC we utilised is considerably longer than current recommendations. However, more recent clinical trials investigating physiologically-based UCC, are reporting much longer cord clamping times (median of ~5 mins) than currently recommended [28]. Similarly, while delayed UCC of one minute is now recommended in infants not requiring resuscitation [11], guidance regarding the timing of prophylactic uterotonic administration is lacking. As a result, there is significant variation in how the third stage of labour is managed worldwide, particularly between midwives and obstetricians [29,30]. Guidelines in Australia, New Zealand, Canada and the United Kingdom recommend maternal oxytocin administration at delivery of the infant’s anterior shoulder [10]. However, there is no level 1 evidence to support this recommendation. On the contrary, a meta-analysis has demonstrated no difference in PPH risk (>500mL) if oxytocin is administered at the beginning or end of third stage [12]. Given, that oxytocin can be safely delayed, our results indicate that oxytocin administration should be delayed until after UCC, but this requires verification in a well-designed clinical trial.

This study was performed in anaesthetised animals which may potentially have blunted the cardiovascular responses (blood pressure, heart rate and blood flow) to the changes in umbilical blood flows. However, we do not believe that this was a significant factor as we have previously demonstrated, using this model, activation of both chemoreceptor and baroreceptor mediated responses [31]. In addition, as the cardiovascular responses reported are very rapid (occurring within 3–4 heart beats), it is highly unlikely that these receptors are involved [1]. Further, uterine contractility (frequency and amplitude) is reduced by volatile anesthetics, including isoflurane [32], suggesting our findings may underestimate the response in non-anaesthetised deliveries.

Whilst our study provides essential insight into the effect of exogenous oxytocin on umbilical cord blood flow and subsequent effect on the physiological transition, it is important to note that the placental structure and umbilical cord are different in humans and sheep. Sheep have placentas consisting of separate cotyledons spread throughout the uterus, rather than a single disc shaped placenta. The ovine umbilical cord contains two umbilical veins and two arteries whereas the human umbilical cord has a single broader diameter vein and two smaller arteries. Nevertheless, our findings in sheep on the role of oxytocin in facilitating placental transfusion are similar to the findings of a clinical trial in humans [26].

In summary, we have demonstrated that maternal oxytocin administration in sheep results in uterine contractions that cause (i) transient reductions in UV and UA flow, (ii) reductions in arterial and cerebral oxygenation and (iii) transient increases in CA pressure and flow in lambs that resemble the increases caused by transient cord occlusions [21]. These findings are consistent with the current understanding for how uterine contractions influence UV and UA flows during labour, but are not consistent with the concept that uterine contractions push blood out of the placenta and into the newborn lambs to affect placental transfusion. Given that oxytocin administration can safely be delayed until after UCC, our results indicate that the question of whether oxytocin should be delayed until after pulmonary ventilation is established and UCC has occurred should be examined clinically. Thus, further research into the timing of oxytocin administration and its impact on the benefits of delayed UCC are required.

## Supporting information

S1 Data Oxytocin data for PLOS. (XLSX)
